# 504. Missed opportunities for HIV Prevention and Treatment in People Hospitalized with Serious Injection Related Infections: The CHOICE+ Cohort

**DOI:** 10.1093/ofid/ofae631.156

**Published:** 2025-01-29

**Authors:** Nicole Bryan, Edward C Traver, Sarah Kattakuzhy, Joseph E Carpenter, Jillian S Catalanotti, Sumitha Raman, Ayako W Fujita, Irene Kuo, Alaina Steck, Elana S Rosenthal, Becky Reece

**Affiliations:** West Virginia University, Morgantown, West Virginia; University of Maryland School of Medicine, Baltimore, MD; Institute for Human Virology (IHV), University of Maryland School of Medicine, Baltimore, Maryland; Emory University School of Medicine, Atlanta, Georgia; The George Washington University of Medicine and Health Sciences, Washington, District of Columbia; George Washington University, Washington, District of Columbia; Emory University, Atlanta, Georgia; George Washington University Milken Institute School of Public Health, Washington, District of Columbia; Emory University, Atlanta, Georgia; Institute for Human Virology (IHV), University of Maryland School of Medicine, Baltimore, Maryland; West Virginia University, Morgantown, West Virginia

## Abstract

**Background:**

Harm reduction for persons who inject drugs (PWID) has many components, including HIV testing and Pre-exposure Prophylaxis (PrEP). However, despite the increasing evidence of effectiveness, PrEP remains highly underutilized in these patients at high-risk for infection and subsequent transmission risk to others. Our aim is to identify the gaps in HIV prevention care among a hospitalized PWID population.

**Table 1:**
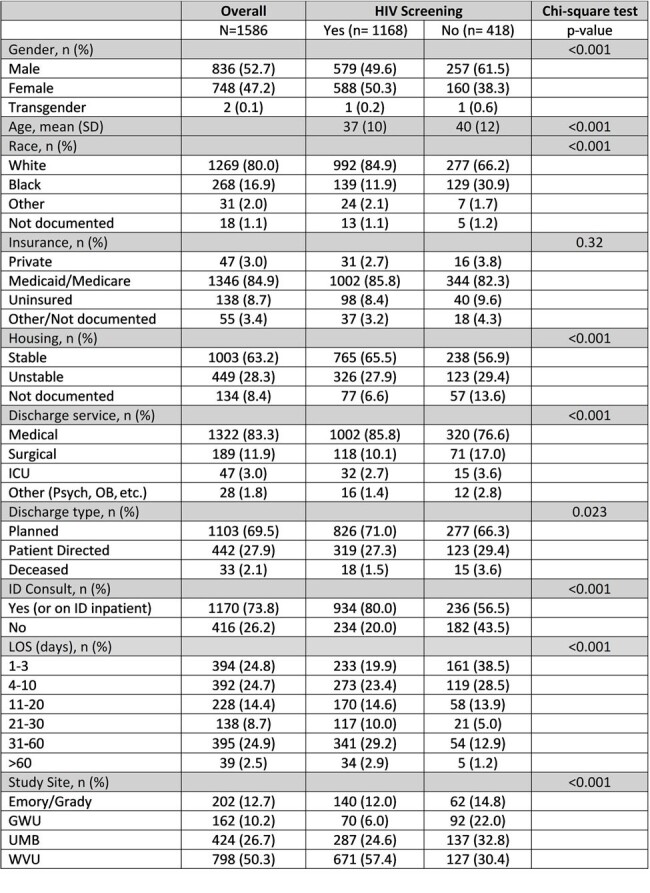
Demographics and Clinical Characteristics

**Methods:**

CHOICE+ is a multisite retrospective cohort study of adults hospitalized with infectious complications of active injection opioid use between 1/1/2018 and 3/31/2022 at four US medical centers. Data were collected by abstraction of the electronic medical record and analyzed by chi-square and multivariate logistic regression.

**Table 2:**
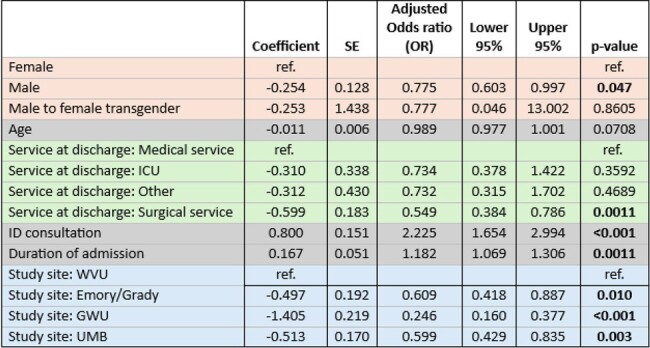
Multivariate Analysis of Factors Associated with HIV Screening

**Results:**

1168 (74%) adults with unknown HIV status underwent HIV screening during sentinel admission. For risk comparison, 48% of this cohort had active HCV infection. HIV screening was less likely to occur in PWID who were male (69% vs 78% females), Black (51% vs 78% White), hospitalized < 3 days (56%), on non-medical services (62% vs 75%), and without ID consult (56% vs 79%). Only 3 participants were referred for PrEP, and only 4 initiated PrEP within 1 year of discharge. Additionally, nearly half (49%) of this HIV (-) cohort did not have follow up HIV screening. There were 79 HIV (+) individuals: 66 known HIV (+) and 13 new diagnoses. Forty-five (57%) were on anti-retroviral therapy during hospitalization, 28 (35%) had CD4 < 200, 47 (59%) had detectable viral load, and 49 (62%) were referred for HIV care. However, only 37% were confirmed linked to care. Of those in care, 59% were virally suppressed within 12 months.

**Table 3:**
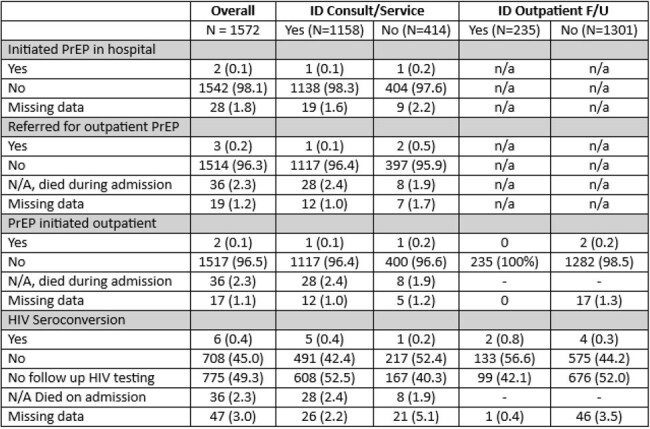
HIV Prevention Outcomes among HIV negative group within 12 months

**Conclusion:**

In a cohort of high-risk PWID hospitalized with infectious complications of substance use, there were low rates of HIV screening, PrEP initiation, and referrals for treatment in HIV (-) individuals. The lack of PrEP initiation in this study is unsurprising; however, the absence of referring patients suggests a lack of awareness of increased PrEP availability. More concerning are the gaps in HIV screening among this high-risk cohort. Clinicians must capitalize on opportunities to provide this population with tools to reduce their risks, which includes HIV screening and PrEP.

**Figure 1:**
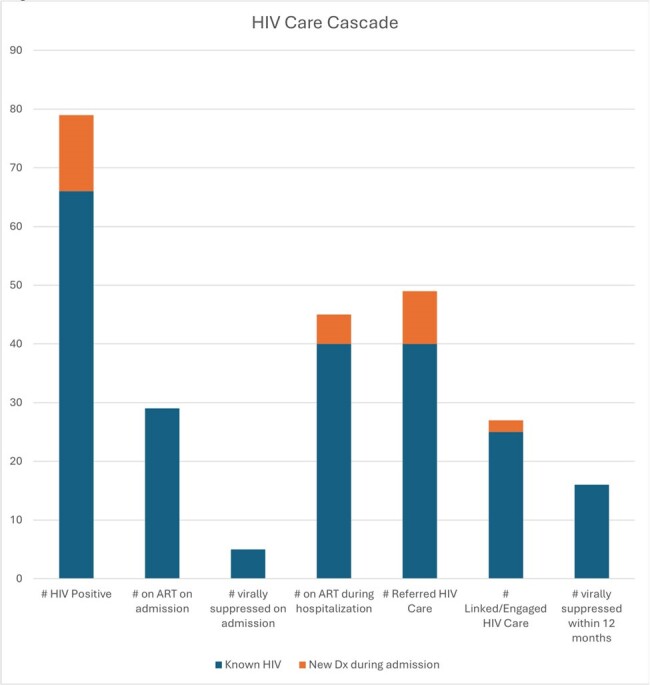
HIV Care Cascade

**Disclosures:**

**Elana S. Rosenthal, MD**, Gilead Sciences: Grant/Research Support|Merck: Grant/Research Support

